# Stability and magnetically induced heating behavior of lipid-coated Fe_3_O_4_ nanoparticles

**DOI:** 10.1186/1556-276X-8-426

**Published:** 2013-10-17

**Authors:** Ayat A Allam, Md Ehsan Sadat, Sarah J Potter, David B Mast, Dina F Mohamed, Fawzia S Habib, Giovanni M Pauletti

**Affiliations:** 1 James L. Winkle College of Pharmacy, University of Cincinnati, Cincinnati, OH 45267, USA; 2 Department of Physics, University of Cincinnati, Cincinnati, OH 45221, USA; 3 Faculty of Pharmacy, Assiut University, 71515 Assiut, Arab Republic of Egypt

**Keywords:** SPION, Magnetic field generator, Hyperthermia, Phospholipid, Thermoresponsive, Colloid

## Abstract

Magnetic nanoparticles that are currently explored for various biomedical applications exhibit a high propensity to minimize total surface energy through aggregation. This study introduces a unique, thermoresponsive nanocomposite design demonstrating substantial colloidal stability of superparamagnetic Fe_3_O_4_ nanoparticles (SPIONs) due to a surface-immobilized lipid layer. Lipid coating was accomplished in different buffer systems, pH 7.4, using an equimolar mixture of 1,2-dipalmitoyl-*sn*-glycero-3-phosphocholine (DPPC) and l-α-dipalmitoylphosphatidyl glycerol (DPPG). Particle size and zeta potential were measured by dynamic laser light scattering. Heating behavior within an alternating magnetic field was compared between the commercial MFG-1000 magnetic field generator at 7 mT (1 MHz) and an experimental, laboratory-made magnetic hyperthermia system at 16.6 mT (13.7 MHz). The results revealed that product quality of lipid-coated SPIONs was significantly dependent on the colloidal stability of uncoated SPIONs during the coating process. Greatest stability was achieved at 0.02 mg/mL in citrate buffer (mean diameter = 80.0 ± 1.7 nm; zeta potential = -47.1 ± 2.6 mV). Surface immobilization of an equimolar DPPC/DPPG layer effectively reduced the impact of buffer components on particle aggregation. Most stable suspensions of lipid-coated nanoparticles were obtained at 0.02 mg/mL in citrate buffer (mean diameter = 179.3 ± 13.9 nm; zeta potential = -19.1 ± 2.3 mV). The configuration of the magnetic field generator significantly affected the heating properties of fabricated SPIONs. Heating rates of uncoated nanoparticles were substantially dependent on buffer composition but less influenced by particle concentration. In contrast, thermal behavior of lipid-coated nanoparticles within an alternating magnetic field was less influenced by suspension vehicle but dramatically more sensitive to particle concentration. These results underline the advantages of lipid-coated SPIONs on colloidal stability without compromising magnetically induced hyperthermia properties. Since phospholipids are biocompatible, these unique lipid-coated Fe_3_O_4_ nanoparticles offer exciting opportunities as thermoresponsive drug delivery carriers for targeted, stimulus-induced therapeutic interventions.

**PACS:**

7550Mw; 7575Cd; 8185Qr

## Background

The use of nanosized colloids offers exciting new opportunities for biomedical applications as they have the potential to overcome significant limitations associated with therapeutic drugs (e.g., physical, chemical, or biochemical instability). In addition, encapsulation of pharmacologically active agents into such nanocarriers allows for spatial and temporal control of drug release, which can significantly improve clinical effects (e.g., controlled and targeted delivery) [1,2].

Superparamagnetic Fe_3_O_4_ nanoparticles (SPIONs) are explored as novel drug delivery systems as their orientation within a magnetic field offers new opportunities to manipulate accumulation and/or drug release in desired target tissues by an externally applied magnetic field [3]*.* Similar to other biomedical applications of SPIONs, including magnetic resonance imaging, biosensing, and cell separation, clinical development critically depends on efficient magnetization and favorable pharmacokinetic properties that minimize clearance by the reticuloendothelial system. It is generally accepted that nanoparticles with hydrophilic surfaces and those less than 200 nm in diameter are compliant with these desired specifications [4,5]*.*

The large surface-to-volume ratio of small magnetic nanoparticles increases surface energy and, thus, enhancing particle aggregation. As a consequence, chemical reactivity decreases, magnetic properties deteriorate, and clearance within a biological system increases [6-9]. Particle stability in an aqueous vehicle can be augmented by electrostatic repulsion using charged surface coatings and/or surface-associated ions, including OH^-^, H_3_O^+^, or buffer ions [10]. The ability to absorb and convert electromagnetic energy into heat distinguishes SPIONs from other nanoassemblies. As heat or 'hyperthermia’ sensitizes living cells to apoptotic stimuli, this unique feature of SPIONs appears specifically beneficial in cancer therapy where temperatures between 40°C and 45°C have been demonstrated to synergistically enhance or potentiate chemotherapy and radiation efficacy [11,12]. Hyperthermia generated by SPIONs following exposure to an alternating magnetic field arises from energy loss associated with oscillation and Néel/Brownian relaxation of the nanoparticle magnetic moment [13]. Stimulus-induced heat generation can also be utilized to control dissociation of a therapeutic moiety from a thermoresponsive carrier that undergoes reversible volume or sol-gel phase transition within a desired range of 37°C to 45°C [14-16]. Previously, our laboratory described a novel phospholipid/Fe_3_O_4_ nanocomposite designed for stimulus-controlled release of an encapsulated payload via magnetically induced hyperthermia [12]. These results demonstrated the feasibility of immobilizing a 2- to 3-nm-thick layer of 1,2-dipalmitoyl-*sn*-glycero-3-phosphocholine (DPPC) on the surface of SPIONs via high affinity avidin/biotin interactions without negatively affecting magnetically induced heating properties. However, moderate surface charge (zeta potential -5.0 ± 3.0 mV) afforded by the zwitterionic but charge-neutral phospholipid assembly resulted in limited colloidal stability, which rapidly led to particle aggregation into the micrometer range [12].

The aim of the present study was to explore the impact of a modified phospholipid composition and different fabrication parameters during the lipid coating process on colloidal stability of these thermoresponsive nanocomposites. In addition, the concentration-dependent heating behavior of these nanoassemblies was compared using two magnetic field generators of different designs. Surface immobilization of an equimolar mixture of DPPC and l-α-dipalmitoylphosphatidyl glycerol (DPPG) on SPIONs significantly increased colloidal stability of these nanocomposites in physiological buffer systems. Exposure to an alternating magnetic field rapidly increased the temperature of the surrounding vehicle as a consequence of magnetically induced hyperthermia. Heating rates were dependent on particle concentration, suspension vehicle, and magnetic field generator design. These results underline the importance of standardized *in vitro* assessment of SPIONs for magnetically induced hyperthermia applications in order to effectively facilitate clinical development of these promising nanocarriers.

## Methods

### Fe_3_O_4_ nanoparticles

SPIONs were synthesized following a previously published coprecipitation method [17]. Briefly, 4.44 g of FeCl_3_·6H_2_O and 1.73 g of FeCl_2_·4H_2_O (Thermo-Fischer Scientific, Pittsburgh, PA, USA) were dissolved in deionized water at a molar ratio of 1:2. Temperature was increased to 70°C while stirring under N_2_ protection before 20 mL of an aqueous 0.5 M NaOH solution was added under continuous stirring. Precipitation was completed after 30 min at 90°C, and SPIONs were collected by magnetic separation following three washes with deionized water.

### Fabrication of lipid-coated Fe_3_O_4_ nanoparticles

A DPPC/DPPG (50:50, mol/mol) lipid coat was immobilized on the surface of SPIONs via high-affinity avidin/biotin interactions as described previously by this laboratory [12]. For a standard fabrication batch, 1 mL of Fe_3_O_4_ nanoparticles suspended at 0.024 mg/mL in citrate buffer, pH 7.4, was incubated with 0.05 mg/mL of avidin at 4°C for 24 h. Excess avidin was removed by three consecutive wash cycles using the same citrate buffer. In a separate 1.5 mL microcentrifuge tube, 95 μL of an equimolar DPPC/DPPG mixture (NOF America, White Plains, NY, USA) prepared in CHCl_3_ was combined with 5 μL of 0.6 mM DSPE-PEG2000-biotin (Avanti Polar Lipids, Alabaster, AL, USA) solution prepared in the same organic solvent. CHCl_3_ was removed under vacuum forming a dry phospholipid film along the centrifuge tube wall. Affinity-stabilized immobilization of a phospholipid layer on avidin-coated SPIONs was induced at room temperature by a 15-min continuous exposure to ultrasonic waves (60 Hz) followed by an additional stabilization period of 30 min at 4°C. Phospholipid-modified Fe_3_O_4_ nanoparticles were washed three times with the buffer solution of interest before used for experiments.

### Physicochemical particle properties

Particle size distribution and electrokinetic potential of uncoated and lipid-coated SPIONs were determined by dynamic laser light scattering (DLS) using the Zetasizer Nano-ZS (Malvern Instruments, Worcestershire, UK) equipped with a 4-mW helium/neon laser (*λ* = 633 nm) and a thermoelectric temperature controller. Particle suspensions prepared in different buffer solutions were preincubated at 25°C for 5 min before each measurement. Particle size values reported in this study correspond to hydrodynamic diameters.

### Magnetically induced hyperthermia

**Figure 1:**
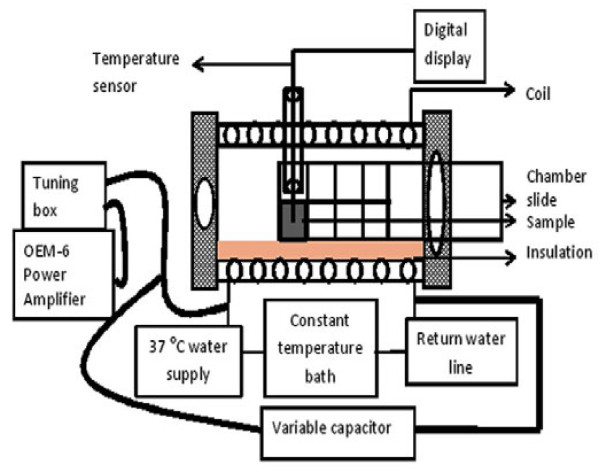
Schematic design of experimental magnetic hyperthermia system (MHS).

Thermal properties of lipid-coated and uncoated control SPIONs were assessed under various conditions following exposure to an alternating magnetic field using the commercial MFG-1000 (lmplementa Hebe, Lund, Sweden) and an experimental magnetic hyperthermia system (MHS) built in our laboratory. Figure 1 shows a schematic diagram of the laboratory-made MHS. It consists of a 10-turn copper coil wrapped around a cylindrical G-10 tube to generate the magnetic field, a connection to a recirculating waterbath that allows control of the environmental temperature inside the coil, and an optical sensor to monitor sample temperature. Styrofoam provides insulation between the coil and the sample. An OEM-6 radio frequency power amplifier operated at 13.56 MHz was used to generate the AC magnetic field. The magnetic field generated in the coil was determined using two turns of a 2-mm magnet wire. For experiments with the MFG-1000, 200 μL of SPION suspension were transferred into a thin-wall PCR tube and repeatedly exposed for 90 s to a 7.0-mT magnetic field alternating at a frequency of 1.0 MHz. Each magnetic pulse was separated by a period of 15 s without a magnetic field to record temperature of the aqueous vehicle using a thermocouple wire [12]. For experiments with the MHS, 300 μL of SPION suspension was filled into one chamber of a Lab-Tek^®^ 8-well chamber slide™ system (Thermo-Fisher Scientific, Pittsburgh, PA, USA) that was subsequently placed inside the copper coil equilibrated at 37°C.

### Statistical analysis

Experiments were performed in triplicate unless otherwise noted. Statistical assessment of differences between experimental groups was performed by one-way ANOVA or two-sided Student's *t* test for pairwise comparison. A probability value of *p* < 0.05 was considered statistically significant (GraphPad Prism 6.0, GraphPad, San Diego, CA, USA).

## Results and discussion

### Fabrications of lipid-coated Fe_3_O_4_ nanoparticles

Thermoresponsive, lipid-coated nanoparticles were fabricated by anchoring a phospholipid bilayer to avidin-coated SPIONs via high-affinity biotin interactions. Previously, this procedure was successfully used to immobilize phospholipid bilayers of different charges on spherical silica substrates [18]. Critical for this fabrication technology is efficient dispersion of SPIONs during the avidin coating process as the lipid components spontaneously encapsulate the avidin-coated 'core’ during the rehydration of the dried film. If this fabrication process is not carefully optimized, avidin-coated particle aggregates will lead to thermoresponsive nanocomposites exhibiting unfavorable particle sizes >200 nm. Fundamentally, adsorption of avidin onto the polar iron oxide surface is facilitated by ionic interactions and enhanced by strong hydrogen bonds [19]. To identify the most suitable fabrication parameters that allow effective avidin coating of highly dispersed SPIONs, particle size distribution and zeta potential of uncoated Fe_3_O_4_ nanoparticles dispersed at 0.02 to 1.0 mg/mL in different buffer systems were measured by DLS. The results summarized in Table 1 consistently demonstrate greater aggregation propensity of SPIONs when particle concentration increases. Irrespective of suspension vehicle, the mean hydrodynamic diameter increased from 0.02 to 0.24 and 1.0 mg/mL, respectively. It is predicted that more frequent collisions at higher particle density overcome weak repulsive surface charges allowing aggregates to be formed, which are stabilized by attractive cohesive forces [20,21]. Metal oxide surfaces can adsorb and/or desorb hydrogen ions as a function of environmental pH. These surface charges interact with electrolytes that are present in the suspension vehicle forming a 'cloud’ of equal but opposite charge, which is commonly known as electrical double layer. At physiological pH 7.4, the Fe_3_O_4_ surface is positively charged facilitating ion pairing with negatively charged counter ions [22]. Zeta potential values measured for uncoated SPIONs in different suspension vehicles demonstrated a dramatic impact of charged buffer ions on the diffuse layer capacitance. This effect is further augmented by an increased particle concentration facilitating significant aggregation. The results from these experiments imply that surface adsorption of trivalent citrate ions most effectively protect SPIONs from aggregation. Even at a concentration of 1.0 mg/mL, the mean particle size was significantly smaller than that measured for the same colloid at a 50-fold lower concentration in Hanks' balanced salt solution (HBSS) or phosphate buffered saline (PBS). Zeta potential values in excess of -32.4 mV implied strong electrostatic repulsion due to surface-associated, fully ionized citrate ions [23]. To fabricate lipid-coated Fe_3_O_4_ nanoparticles at the desired target size range of <200 nm, the avidin coating step was performed at 0.02 mg/mL in citrate buffer, which afforded particle populations with a mean hydrodynamic diameter of approximately 80 nm. The lipid composition was selected with the objective to fabricate thermoresponsive colloids that exhibit a transition temperature consistent with clinical hyperthermia applications (40°C to 45°C) [11].

**Table 1:** Physicochemical properties of uncoated and lipid-coated SPIONs in different buffer solutions at pH 7.4

**Buffer system**	**Particle concentration (mg/mL)**	**Particle size (nm)**		**Zeta potential (mV)**	
		**Uncoated SPIONs**	**Lipid-coated SPIONs**	**Uncoated SPIONs**	**Lipid-coated SPIONs**
	1.0	520.0 ± 45.4	651.6 ± 25.3	-32.4 ± 1.0	-11.9 ± 1.4
Citrate, pH 7.4	0.24	286.6 ± 25.4	460.3 ± 15.4	-40.7 ± 1.4	-15.6 ± 1.4
	0.02	80.0 ± 1.7*	179.3 ± 35.0**	-47.1 ± 2.6*	-19.1 ± 1.3**
	1.0	1860.0 ± 180.9^a^	2422.0 ± 223.5^a^	-11.2 ± 1.0	-4.5 ± 0.9
HBSS, pH 7.4	0.24	1255.0 ± 35.2^a^	1560.0 ± 135.2^a^	-12.3 ± 1.1	-5.5 ± 1.0
	0.02	580.0 ± 8.5	193.5 ± 32.6**	-23.3 ± 0.8	-7.4 ± 1.4
	1.0	2800.0 ± 320.4^a^	2990.0 ± 412.5^a^	-10.3 ± 0.5	-2.2 ± 0.6
PBS, pH 7.4	0.24	2214.0 ± 45.3^a^	2500.0 ± 245.3^a^	-10.8 ± 1.0	-3.4 ± 1.1
	0.02	931.0 ± 4.5	229.9 ± 12.42**	-22.5 ± 0.8	-5.2 ± 1.6

Data are represented as mean ± SD (*n* = 4).

^a^Value outside qualification range of Zetasizer Nano-ZS.

*Significantly different from uncoated control SPIONs (*p* < 0.05).

**Significantly different from lipid-coated SPIONs (*p* < 0.05).

Earlier experiments performed in our laboratory with DPPC-coated SPIONs revealed limited colloidal stability in physiological buffer systems due to low surface charge (zeta potential -5.0 mV) [12]. DPPG is a negatively charged phosphatidyl glycerol with the same transition temperature as DPPC (i.e., 41°C). Stability of liposomes prepared with mixtures of these two phospholipids has been studied previously, and an equimolar lipid ratio was demonstrated to enhance colloidal stability [24]. When comparing physicochemical properties of lipid-coated SPIONs suspended in different buffer systems (Table 1), it was notable that the mean particle size significantly increased to 179 nm after the coating procedure. It is conceivable that the modified avidin coating protocol using citrate buffer altered the charge distribution at the steric layer, thus augmenting the negative surface charge of avidin-coated SPIONs. With the introduction of the negatively charged DPPG into the lipid mixture, charge repulsion may have resulted in less tight association of the lipid layer with the avidin-coated Fe_3_O_4_ surface. Further assessment of the nanoassembly using high-resolution transmission electron microscopy (HRTEM) and atomic force microscopy could provide additional experimental support for this hypothesis. Nevertheless, it is relevant to emphasize that DLS measurements are performed in the presence of a liquid suspension vehicle (e.g., citrate buffer) and determine hydrodynamic particle size distributions. HRTEM requires dry samples and may result in different quantitative size information due to the absence of a surface-associated hydration layer. The incorporation of a 50% molar ratio of DPPG into the lipid layer effectively augmented the negative surface charge of the lipid coat from -5.0 [12] to -19.1 mV. The enhanced negative charge associated with the nanoparticle surface is expected to increase colloidal stability of the suspension. Furthermore, it is predicted that this favorable zeta potential reduces surface adsorption of serum components such as proteins and lipoproteins [25]. Ultimately, these improved physicochemical properties of lipid-coated SPIONs may significantly increase biological circulation time after systemic administration allowing more effective delivery of therapeutic payload to desired target cells.

### Magnetically induced hyperthermia

**Figure 2:**
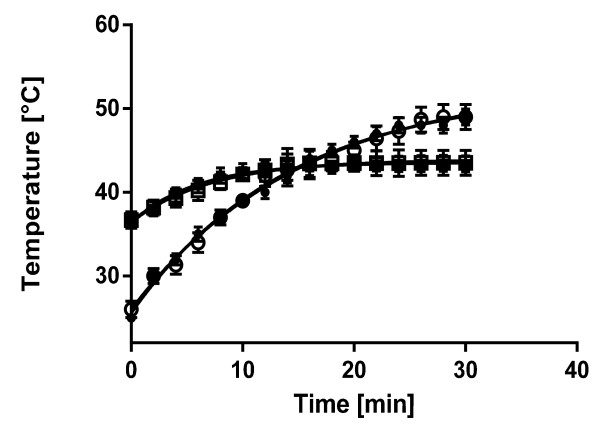
**Heating behavior of uncoated and lipid-coated SPIONs within an alternating magnetic field.** Uncoated (open symbols) and lipid-coated (closed symbols) Fe_3_O_4_ nanoparticles suspended at 0.02 mg/mL in citrate buffer, pH 7.4, were exposed in the MGS-1000 to an alternating magnetic field of 7.0 mT at 1.0 MHz (circles) and in the MHS to 16.6 mT at 13.6 MHz (squares). Temperature of suspension vehicle was recorded using an optical fiber probe. Data are shown as mean ± SD (*n* = 3).

The objective of immobilizing a phospholipid layer onto the surface of SPIONs was to fabricate a thermoreponsive nanoassembly that facilitates stimulus-induced release of a lipid-encapsulated payload following exposure to a localized alternating magnetic field. Heating behavior of uncoated and lipid-coated SPIONs was first assessed in the MFG-1000, which represents a user-friendly commercial device for the assessment of hyperthermia up to 7.0 mT at 1.0 MHz. It allows simple measurements using 200-μL PCR tubes or glass slides. However, this device has limited suitability for cell-based experiments and cannot be used for preclinical animal experiments. Therefore, it was of interest to compare heating behaviors of these SPIONs in the MFG-1000 with results from an experimental MHS built in our laboratory that was designed to explore the magnetically induced hyperthermia effect on biological systems, including adherent cell lines and small animals such as mice and rats. Figure 2 compares time-dependent temperature profiles recorded upon exposure of lipid-coated SPIONs at a concentration of 0.02 mg/mL in citrate buffer, pH 7.4, to a 7-mT magnetic field alternating at 1.0 MHz (MFG-1000) and a 16.6-mT magnetic field at 13.6 MHz (MHS). In both devices, the thermal behavior of Fe_3_O_4_ nanoparticles was not significantly affected by the presence of the lipid layers as demonstrated by the superimposable temperature profiles. This implies deposition of a relatively thin lipid layer around the Fe_3_O_4_ core that did not dramatically impact oscillation and relaxation of these superparamagnetic nanocomposites. This conclusion is further supported by the absence of significant change in temperature profile around the anticipated melting temperature of 41°C. Review of hyperthermia kinetics, however, suggests that the design of the magnetic field generator significantly impacts conversion of electromagnetic energy into heat. Most notably, heating profiles generated in the MFG-1000 begin at room temperature and appear to plateau after 30 min around 50°C. In contrast, temperature profiles measured in MHS, which was maintained at 37°C prior to initiation of the alternating magnetic field, revealed a maximum temperature of only 43°C despite a two-fold stronger magnetic field. It is hypothesized that the large space in the experimental device designed to accommodate test samples up to small animals acts as an effective heat sink preventing temperature increases above 43°C. It remains to be explored whether the apparent steady-state temperature of 43°C can be maintained in preclinical animals without the adjustment of the magnetic field. If required, a feedback loop could be engineered into this device that facilitates real-time field adjustments using a coupled sensor circuit. However, the results from this study demonstrate the feasibility of effectively raising the temperature of this magnetic fluid to the clinically relevant hyperthermia range of 40°C to 45°C within 10 min using alternating magnetic fields between 7 and 17 mT.

Heat production by SPIONs following exposure to an alternating magnetic field are consequences of several types of loss processes, including hysteresis as well as Néel and Brownian relaxations [26,27]. Brownian relaxation loss is due to the physical rotation of the particles within the fluid whereas Néel relaxation loss occurs when magnetic moments of individual nanoparticles overcome the energy barrier between easy axis orientations. The time delay between the alignment time and effective relaxation time results in an energy transfer from the SPIONs to the surrounding environment [26,28]. Initial heating rates represent inherent thermal properties of the material tested without system-associated limitations (e.g., device configuration). Table 2 summarizes the results of these kinetic analyses performed with uncoated and lipid-coated SPIONs that were suspended at 0.02 to 1.0 mg/mL in different buffer systems. For uncoated SPIONs dispersed in citrate buffer, initial heating rates were slightly greater for dilute suspensions that were found to exhibit the smallest particle size (see Table 1). The apparently more effective conversion of magnetically induced particle relaxation into thermal energy that was measured in the MFG-1000 may be associated with the small void space around the sample in this device resulting in improved heat transfer. Thermal properties of lipid-coated SPIONs at 0.02 mg/mL in different buffer systems were comparable suggesting limited surface adsorption of buffer components onto lipid-coated nanoparticles which is consistent with the earlier size analysis (see Table 1). Interestingly, initial heating rates of lipid-coated SPIONs significantly increased at greater particle concentration. DLS data revealed a significantly increased hydrodynamic size of lipid-coated particles at greater particle density, which is anticipated to negatively affect the heating properties. Ultrastructural analysis of these nanoassemblies using HRTEM may provide insights into why these larger superparamagnetic particles convert magnetically induced oscillation and relaxation more efficiently into heat. It may be possible that the apparent larger particle size may correspond to encapsulation of several superparamagnetic Fe_3_O_4_ nanoparticles within a semisolid lipid particle that can experience enhanced relaxation loss during temperature-induced sol-gel transition of the lipid phase.

**Table 2:** Initial heating rates of uncoated and lipid-coated SPIONs following exposure to an alternating magnetic field

**Particle concentration/suspension vehicle**	**Initial heating rate (°C/min)**			
	**MFG-1000 at 7.0 mT (1 MHz)**		**MHS at 16.6 mT (13.6 MHz)**	
	**Uncoated SPIONs**	**Lipid-coated SPIONs**	**Uncoated SPIONs**	**Lipid-coated SPIONs**
1 mg/mL (Citrate buffer)	0.88 ± 0.02	1.26 ± 0.03**	0.35 ± 0.01	0.61 ± 0.02**
0.24 mg/mL (Citrate buffer)	0.90 ± 0.02	1.05 ± 0.04	0.36 ± 0.02	0.56 ± 0.01
0.02 mg/mL (Citrate buffer)	0.95 ± 0.03*	0.94 ± 0.02	0.47 ± 0.01*	0.46 ± 0.01
0.02 mg/mL (HBSS)	0.66 ± 0.02	0.94 ± 0.01	0.33 ± 0.01	0.44 ± 0.02
0.02 mg/mL (PBS)	0.55 ± 0.02	0.92 ± 0.02	0.20 ± 0.01	0.43 ± 0.01

Data are shown as mean ± SD (*n* = 3).

*Significantly different from uncoated control SPIONs at 0.02 mg/mL (*p* < 0.05).

**Significantly different from lipid-coated SPIONs at 0.02 mg/mL in citrate buffer (*p* < 0.05).

## Conclusion

The results from this study demonstrate that surface immobilization of an equimolar DPPC/DPPG mixture on SPIONs via high-affinity avidin-biotin interactions increases colloidal stability in the presence of different buffer ions. Citrate buffer, pH 7.4, provides a significant advantage during avidin coating due to efficient colloid dispersion as a consequence of negative surface charge. Magnetically induced heating properties of uncoated and lipid-coated SPIONs were significantly dependent on the design of the magnetic field generator used. However, therapeutically relevant hyperthermia (>40°C was achieved within 10 min following exposure to an alternative magnetic field between 7 and 17 mT. These results underline that biocompatible, phospholipid-coated SPIONs offer exciting opportunities as thermoresponsive drug delivery carriers for targeted, stimulus-induced therapeutic interventions.

## Abbreviations

DLS: Dynamic laser light scattering; DPPC: 1,2-dipalmitoyl-*sn*-glycero-3-phosphocholine; DPPG: l-α-dipalmitoylphosphatidyl glycerol; HRTEM: High-resolution transmission electron microscopy; MHS: Magnetic hyperthermia system; SPION: Superparamagnetic iron oxide nanoparticles.

## Competing interests

The authors declare that they have no competing interests.

## Authors' contributions

AAA carried out the fabrication, physicochemical characterization, and magnetically induced heating assessment of lipid-coated SPIONs. MES built the experimental MHS and participated in magnetically induced heating assessment. SJP assisted in the fabrication and physicochemical characterization of lipid-coated SPIONs and helped in the drafting of the manuscript. DBM conceived the design of the MHS and participated in its construction. DFM and FSH participated in the design of this study. GMP conceived the study, coordinated experimental designs, and helped drafting the manuscript. All authors read and approved the final manuscript.
